# Supplementary material for: Association of Vitamin D Supplementation with Cardiovascular Events: A Systematic Review and Meta-Analysis
Source: Nutrients. 2022 Jul 30;14(15):3158. doi: 10.3390/nu14153158 (PMC9370368; doi:10.3390/nu14153158)
Supplement: Supplementary file 1 [file nutrients-14-03158-s001.zip › nutrients-1831010-supplementary.pdf]

Supplemental e-material

Table S1: Search strategy

Table S2: Sensitivity analyses

Figure S1: Study selection process

Figure S2: Risk of bias summary

Figure S3: Risk of bias graph

Figure S4: Funnel plot for Cardiovascular mortality

Figure S5: Funnel plot for Stroke

Figure S6: Funnel plot for Myocardial infarction

Figure S7: Funnel plot for Cardiovascular events

Figure S8: Funnel plot for Cerebrovascular event

Figure S9: Trial sequential analysis for Cardiovascular mortality, RRR=15%

Figure S10: Trial sequential analysis for Stroke, RRR=15%

Figure S11: Trial sequential analysis for Myocardial infarction, RRR=15%

Figure S12: Trial sequential analysis for Cardiovascular events, RRR=15%

Figure S13: Trial sequential analysis for Cerebrovascular events, RRR=15%

Table S1: Search strategy

**MEDLINE(R)**		
1	exp Vitamin D/ad, ae, pd, tu [Administration & Dosage, Adverse Effects, Pharmacology, Therapeutic Use]	34502
2	((vit-d? or vitamin-d? or dihydroxyvitamin D? or dihydroxy-vitamin D? or hydroxyvitamin D? or hydroxyl-vitamin d? or dihydrotachysterol or colecalciferol or epicalcitriol or oxacalcitriol or alfacalcidol or calcifediol I or calciferol? or calcipotriol or calcitriol or dihydroxycolecalciferol or hydroxycolecalciferol or seocalcitol or tacalcitol or oxavitamin or hydroxycholecalciferol? or calcidiol or calcipotriene or dihydroxycholecalciferol? or dihydroxy-cholecalciferol or cholecalciferol? or ergocalciferol? or epiergocalciferol or dihydroxyergocalciferol or dihydroxy-ergocalciferol or hydroxyl-ergocalciferol or hydroxyergocalciferol or doxercalciferol or hydroxycalciferol or hydroxyl-calciferol or dihydroxy-calciferol or dihydroxycalciferol or dihydrotachysterin or calcamine or ercalcidiol) adj5 (supplement* or therap* or treat* or prevent* or daily or receiv* or regimen or dose? or oral* or intramuscular or inject*)).mp.	25805
3	randomized controlled trial.pt.	565485
4	controlled clinical trial.pt.	94812
5	random*.mp.	1368013
6	placebo.ab.	208941
7	drug therapy.fs.	2478813
8	trial.ab.	524411
9	groups.ab.	2018313
10	or/3-9	5172554
11	exp animals/ not humans.sh.	4996421
12	10 not 11	4757726
13	(1 or 2) and 12	27300
14	limit 13 to "all adult (19 plus years)"	14974
**Embase**		
1	exp vitamin D/ae, ct, ad, cb, cm, cr, do, dt, dl, du, ig, im, na, os, iv, po, pa, pd, li, tp, td [Adverse Drug Reaction, Clinical Trial, Drug Administration, Drug Combination, Drug Comparison, Drug Concentration, Drug Dose, Drug Therapy, Intradermal Drug Administration, Intraduodenal Drug Administration, Intragastric Drug Administration, Intramuscular Drug Administration, Intranasal Drug	48826

	Administration, Intraosseous Drug Administration, Intravenous Drug Administration, Oral Drug Administration, Parenteral Drug Administration, Pharmacology, Sublingual Drug Administration, Topical Drug Administration, Transdermal Drug Administration]	
2	((vit-d? or vitamin-d? or dihydroxyvitamin D? or dihydroxy-vitamin D? or hydroxyvitamin D? or hydroxyl-vitamin d? or dihydrotachysterol or colecalciferol or epicalcitriol or oxacalcitriol or alfacalcidol or calcifediol I or calciferol? or calcipotriol or calcitriol or dihydroxycolecalciferol or hydroxycolecalciferol or seocalcitol or tacalcitol or oxavitamin or hydroxycholecalciferol? or calcidiol or calcipotriene or dihydroxycholecalciferol? or dihydroxy-cholecalciferol or cholecalciferol? or ergocalciferol? or epiergocalciferol or dihydroxyergocalciferol or dihydroxy-ergocalciferol or hydroxyl-ergocalciferol or hydroxyergocalciferol or doxercalciferol or hydroxycalciferol or hydroxyl-calciferol or dihydroxy-calciferol or dihydroxycalciferol or dihydrotachysterin or calcamine or ercalcidiol) adj5 (supplement* or therap* or treat* or prevent* or daily or receiv* or regimen or dose? or oral* or intramuscular or inject*)).mp.	46452
3	randomized controlled trial/	707182
4	crossover procedure/	70209
5	double blind procedure/	194560
6	single blind procedure/	46005
7	(random* or factorial* or crossover* or placebo* or assign* or allocat* or volunteer* or (doubl* adj5 blind*) or (singl* adj5 blind*)).mp.	2941714
8	or/3-7	2941714
9	exp animal/	28509119
10	human/	23421865
11	9 not 10	4389580
12	8 not 11	2718129
13	(1 or 2) and 12	24586
14	limit 13 to (adult <18 to 64 years> or aged <65+ years>)	10953

EBM Reviews - Cochrane Central Register of Controlled Trials

1	exp Vitamin D	5999
2	((vit-d? or vitamin-d? or dihydroxyvitamin D? or dihydroxy-vitamin D? or hydroxyvitamin D? or hydroxyl-vitamin d? or dihydrotachysterol or colecalciferol or epicalcitriol or oxacalcitriol or alfacalcidol or calcifediol I or calciferol? or calcipotriol or calcitriol or dihydroxycolecalciferol or hydroxycolecalciferol or seocalcitol or tacalcitol or oxavitamin or hydroxycholecalciferol? or calcidiol or calcipotriene or dihydroxycholecalciferol? or dihydroxy-cholecalciferol or cholecalciferol? or ergocalciferol? or epiergocalciferol or dihydroxyergocalciferol or dihydroxy-ergocalciferol or hydroxyl-ergocalciferol or hydroxyergocalciferol or doxercalciferol or hydroxycalciferol or hydroxyl-	11658

	calciferol or dihydroxy-calciferol or dihydroxycalciferol or dihydrotachysterin or calcamine or ercalcidiol) adj5 (supplement* or therap* or treat* or prevent* or daily or receiv* or regimen or dose? or oral* or intramuscular or inject*)).mp.	
3	1 or 2	15724

Table S2: Sensitivity analyses

Cardiovascular mortality

Sensitivity analyses	Risk Ratio, 95% CI	I^2	P
Excluding studies with high or unknown risk of bias	1.00 [0.87, 1.15]	0%	0.96
Excluding studies with high or unknown risk of bias of each domain			
Sequence generation	0.96 [0.87, 1.06]	0%	0.42
Allocation concealment	0.96 [0.87, 1.06]	0%	0.42
Blinding of patients and personnel	0.96 [0.87, 1.06]	0%	0.42
Blinding of outcome assessors	0.96 [0.87, 1.06]	0%	0.43
Incomplete outcome data	0.96 [0.87, 1.06]	0%	0.45
Selective reporting	1.00 [0.87, 1.15]	0%	0.96
Other bias	0.96 [0.88, 1.06]	0%	0.46
Excluding quasi-randomized or cluster-randomized trials	0.96 [0.88, 1.06]	0%	0.46
Using random-effect models	0.96 [0.88, 1.06]	0%	0.46

Stroke

Sensitivity analyses	Risk Ratio, 95% CI	I^2	P
Excluding studies with high or unknown risk of bias	0.99 [0.84, 1.16]	0%	0.89
Excluding studies with high or unknown risk of bias of each domain			
Sequence generation	1.04 [0.91, 1.20]	0%	0.53
Allocation concealment	1.04 [0.90, 1.19]	0%	0.61
Blinding of patients and personnel	1.04 [0.91, 1.19]	0%	0.53

Blinding of outcome assessors	1.05 [0.92, 1.20]	0%	0.51
Incomplete outcome data	1.05 [0.92, 1.20]	0%	0.48
Selective reporting	1.01 [0.86, 1.18]	0%	0.92
Other bias	1.04 [0.91, 1.19]	0%	0.52
Excluding quasi-randomized or cluster-randomized trials	1.05 [0.92, 1.20]	0%	0.47
Using random-effect models	1.05 [0.92, 1.20]	0%	0.47

Myocardial infarction

Sensitivity analyses	Risk Ratio, 95% CI	I^2	P
Excluding studies with high or unknown risk of bias	0.95 [0.84, 1.08]	0%	0.44
Excluding studies with high or unknown risk of bias of each domain			
Sequence generation	0.95 [0.85, 1.06]	0%	0.38
Allocation concealment	0.95 [0.85, 1.06]	0%	0.36
Blinding of patients and personnel	0.96 [0.85, 1.07]	0%	0.45
Blinding of outcome assessors	0.96 [0.85, 1.07]	0%	0.97
Incomplete outcome data	0.97 [0.86, 1.08]	0%	0.57
Selective reporting	0.96 [0.85, 1.09]	0%	0.52
Other bias	0.97 [0.86, 1.08]	0%	0.56
Excluding quasi-randomized or cluster-randomized trials	0.96 [0.85, 1.09]	0%	0.81
Using random-effect models	0.97 [0.87, 1.08]	0%	0.59

Total cardiovascular events

Sensitivity analyses	Risk Ratio, 95% CI	I^2	P
Excluding studies with high or unknown risk of bias	0.99 [0.90, 1.09]	34%	0.79
Excluding studies with high or unknown risk of bias of each domain			
Sequence generation	0.99[0.90, 1.09]	27%	0.82
Allocation concealment	0.99[0.90, 1.09]	27%	0.82
Blinding of patients and personnel	0.99[0.90, 1.09]	27%	0.82
Blinding of outcome assessors	0.99[0.90, 1.09]	27%	0.82
Incomplete outcome data	0.99[0.90, 1.09]	27%	0.82
Selective reporting	0.99[0.90, 1.09]	27%	0.82
Other bias	0.99 [0.90, 1.09]	34%	0.79

Excluding quasi-randomized or cluster-randomized trials	0.99[0.90, 1.09]	27%	0.82
Using random-effect models	0.97[0.91, 1.04]	27%	0.43

Cerebrovascular events

Sensitivity analyses	Risk Ratio, 95% CI	I^2	P
Excluding studies with high or unknown risk of bias	0.97 [0.83, 1.14]	0%	0.75
Excluding studies with high or unknown risk of bias of each domain			
Sequence generation	0.99 [0.85, 1.16]	0%	0.93
Allocation concealment	0.98 [0.84, 1.15]	0%	0.94
Blinding of patients and personnel	0.99 [0.85, 1.16]	0%	0.93
Blinding of outcome assessors	0.99 [0.85, 1.16]	0%	0.93
Incomplete outcome data	1.01 [0.86, 1.18]	0%	0.93
Selective reporting	0.99 [0.85, 1.16]	0%	0.93
Other bias	1.00 [0.86, 1.17]	0%	0.99
Excluding quasi-randomized or cluster-randomized trials	1.01 [0.86, 1.18]	0%	0.93
Using random-effect models	1.01 [0.87, 1.18]	0%	0.90

Figure S1: Study selection process

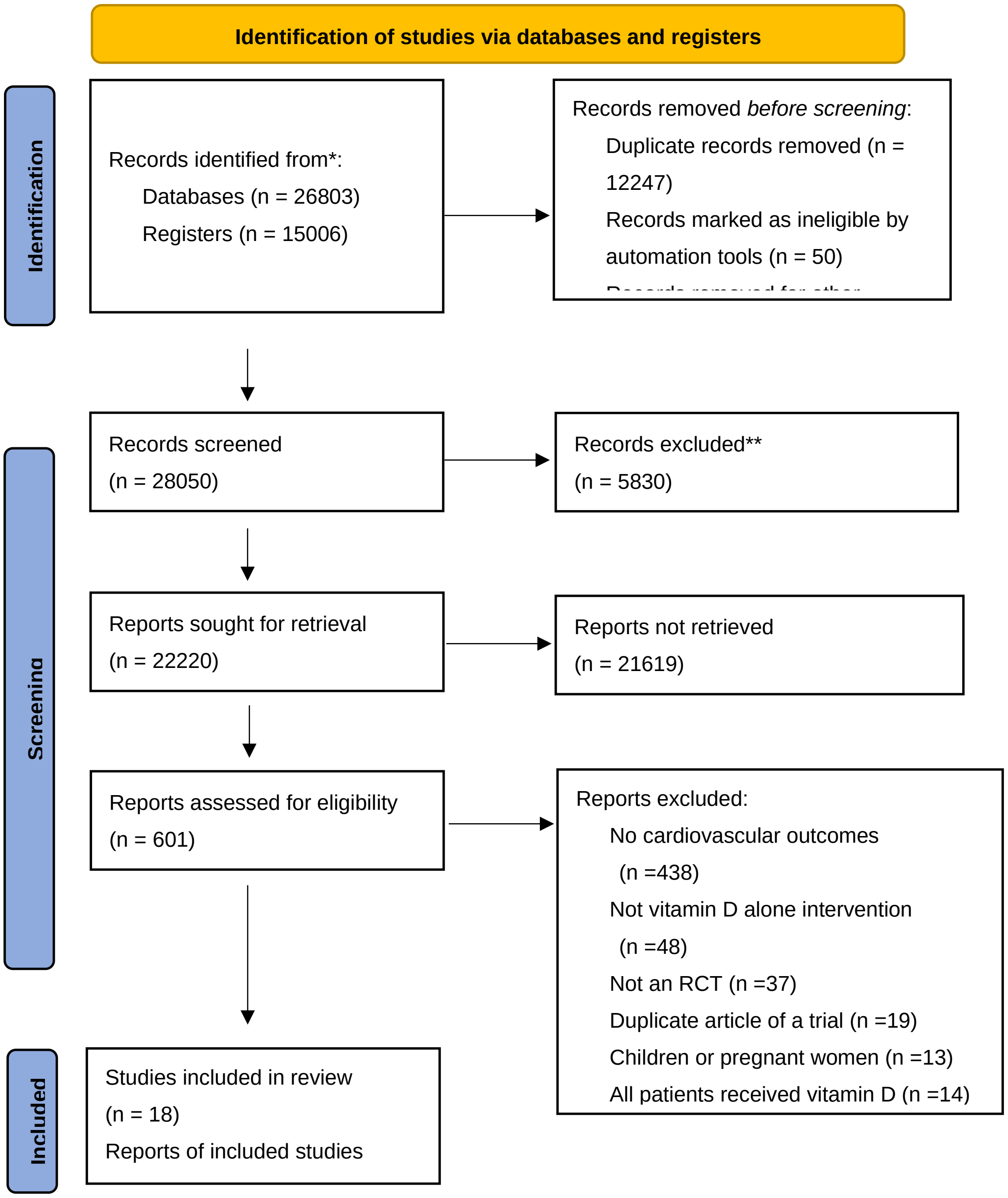

*Consider, if feasible to do so, reporting the number of records identified from each database or register searched (rather than the total number across all databases/registers).

**If automation tools were used, indicate how many records were excluded by a human and how many were excluded by automation tools.

From: Page MJ, McKenzie JE, Bossuyt PM, Boutron I, Hoffmann TC, Mulrow CD, et al. The PRISMA 2020 statement: an updated guideline for reporting systematic reviews. BMJ 2021;372:n71. doi: 10.1136/bmj.n71

For more information, visit: http://www.prisma-statement.org/

Figure S2: Risk of bias summary: review authors' judgements about each risk of bias item for each included study.

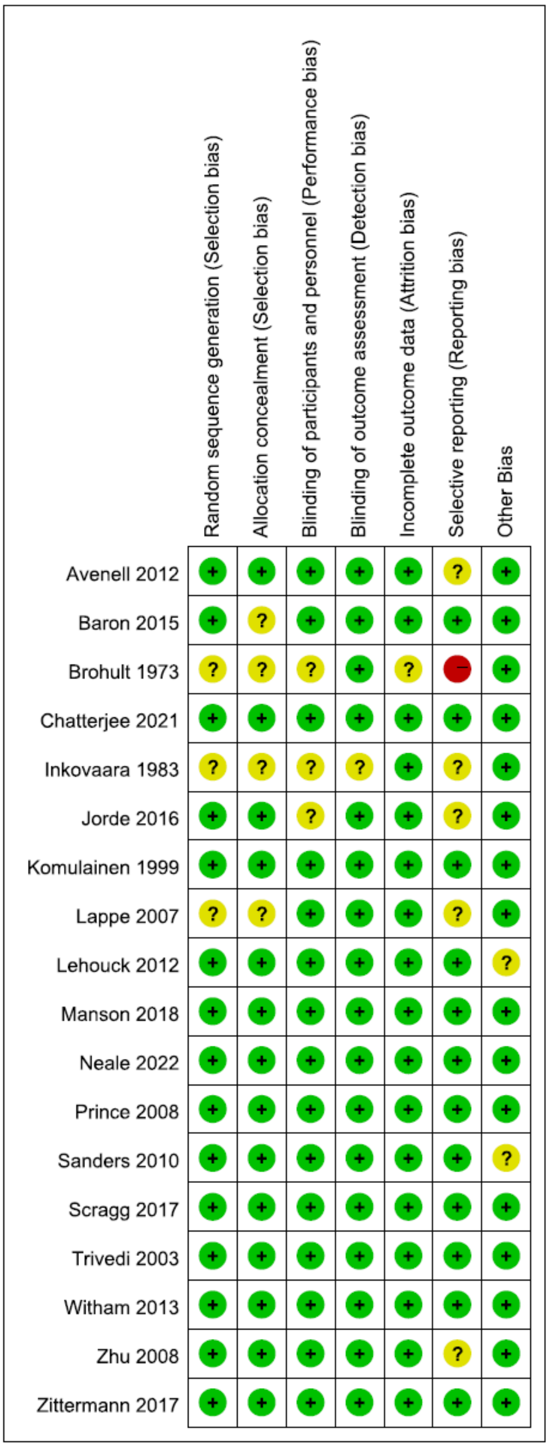

Figure S3: Risk of bias graph: review authors' judgements about each risk of bias item presented as percentages across all included studies

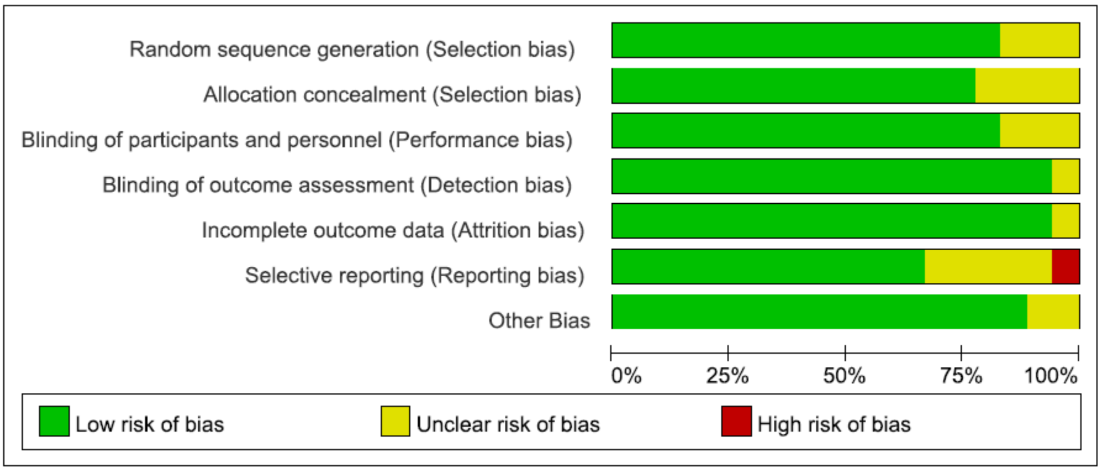

Figure S4: Funnel plot of cardiovascular mortality

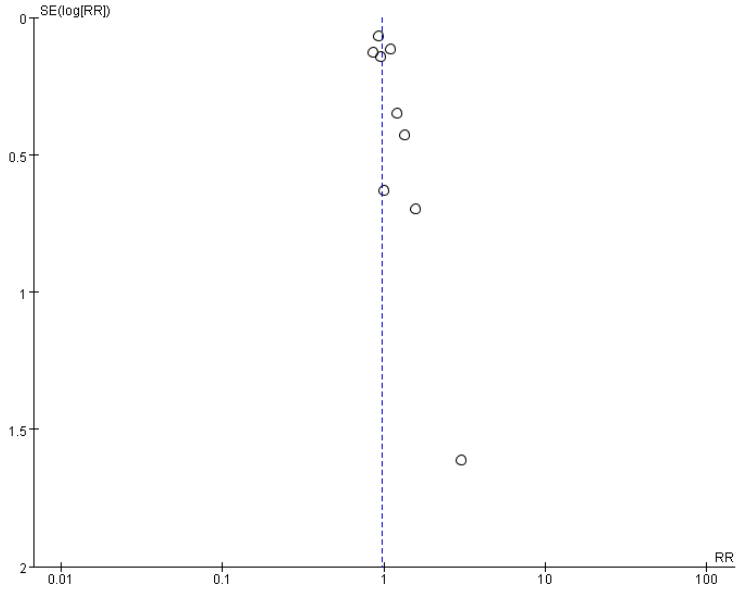

Figure S5: Funnel plot of stroke

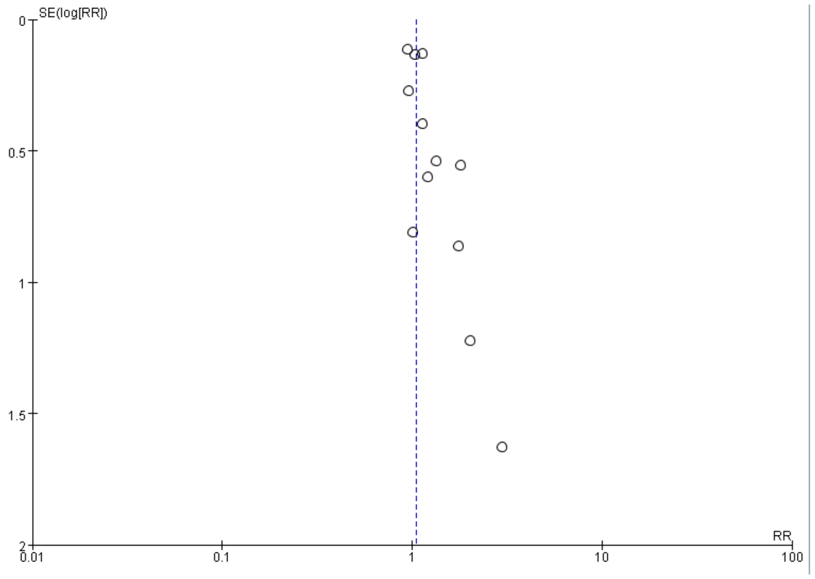

Figure S6: Funnel plot of myocardial infarction

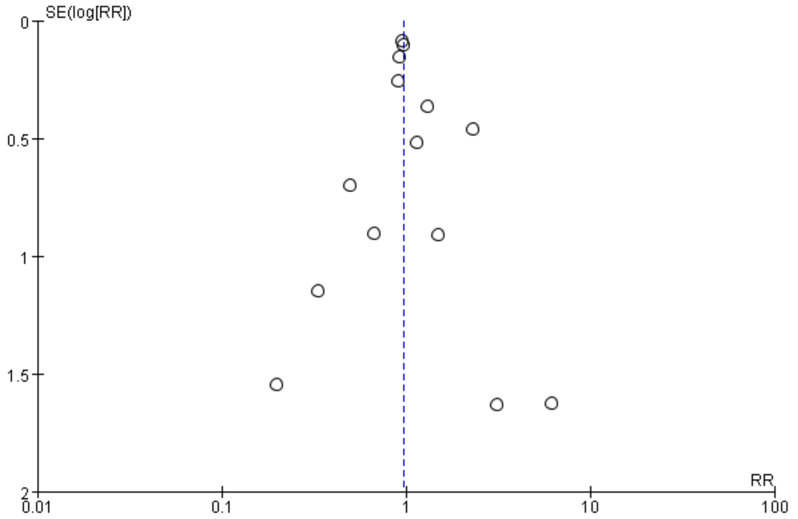

Figure S7: Funnel plot of total cardiovascular events

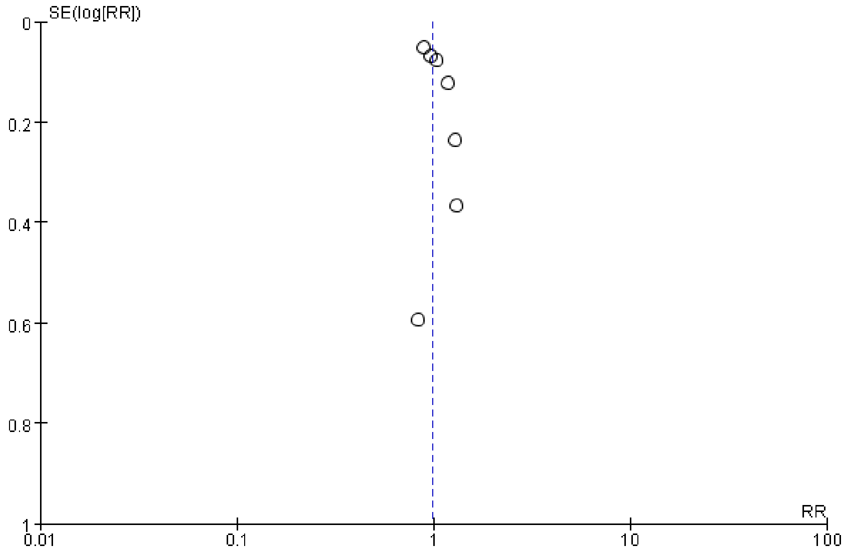

Figure S8: Funnel plot of cerebrovascular events

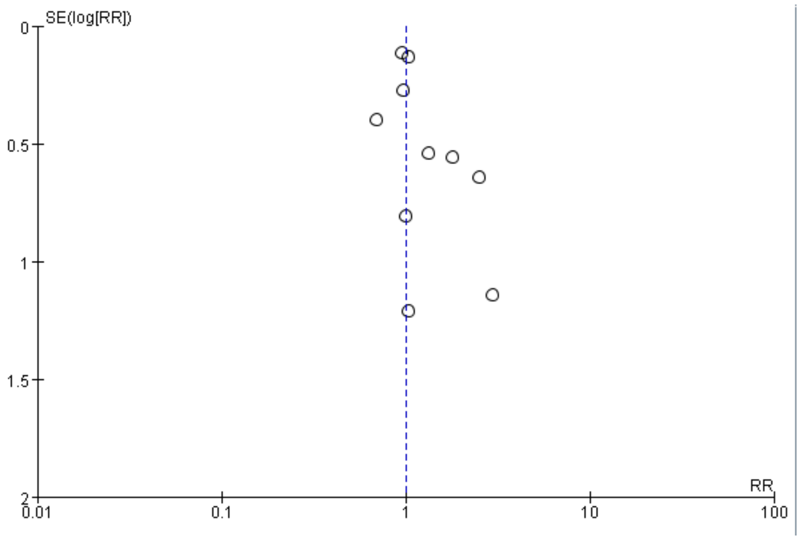

Figure S9 Trial sequential analysis for cardiovascular mortality,

RRR=15%

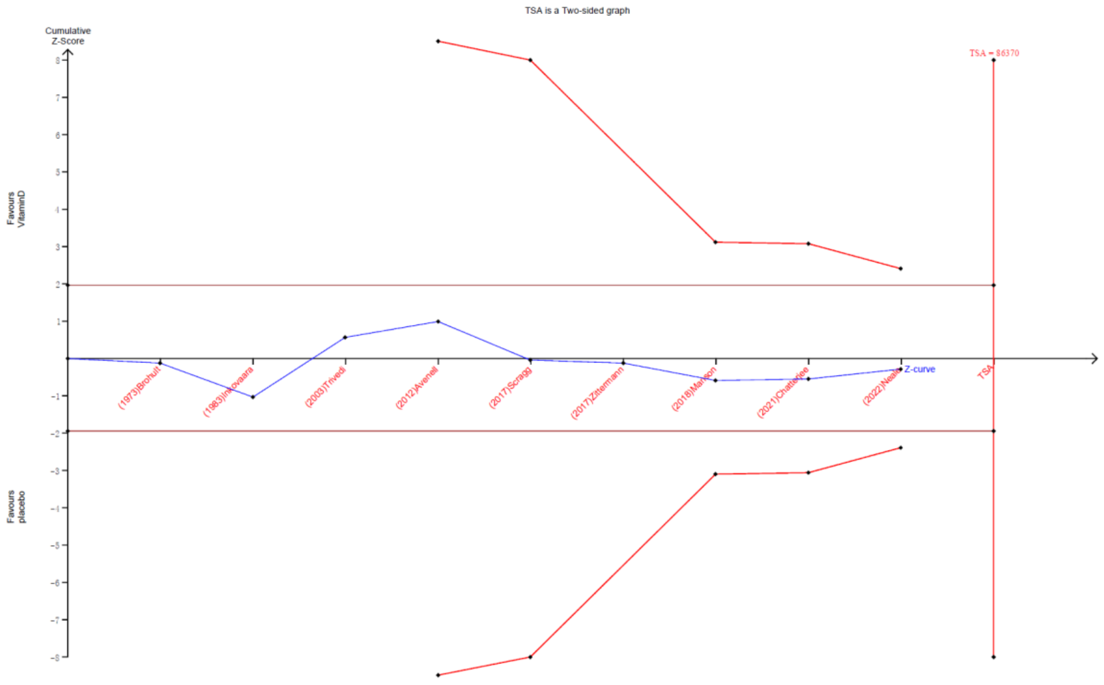

A diversity-adjusted information size (DIS) size of 86370 patients was calculated based on an anticipated relative risk reduction (RRR) of15% (event proportion of 2.4% in the control arm, α=0.05 (two-sided), β=0.20 (power 80%)). The blue cumulative z-curve was constructed using a random-effects model and crossed the boundary for futility.

FigureS10: Trial sequential analysis for stroke, RRR=15%

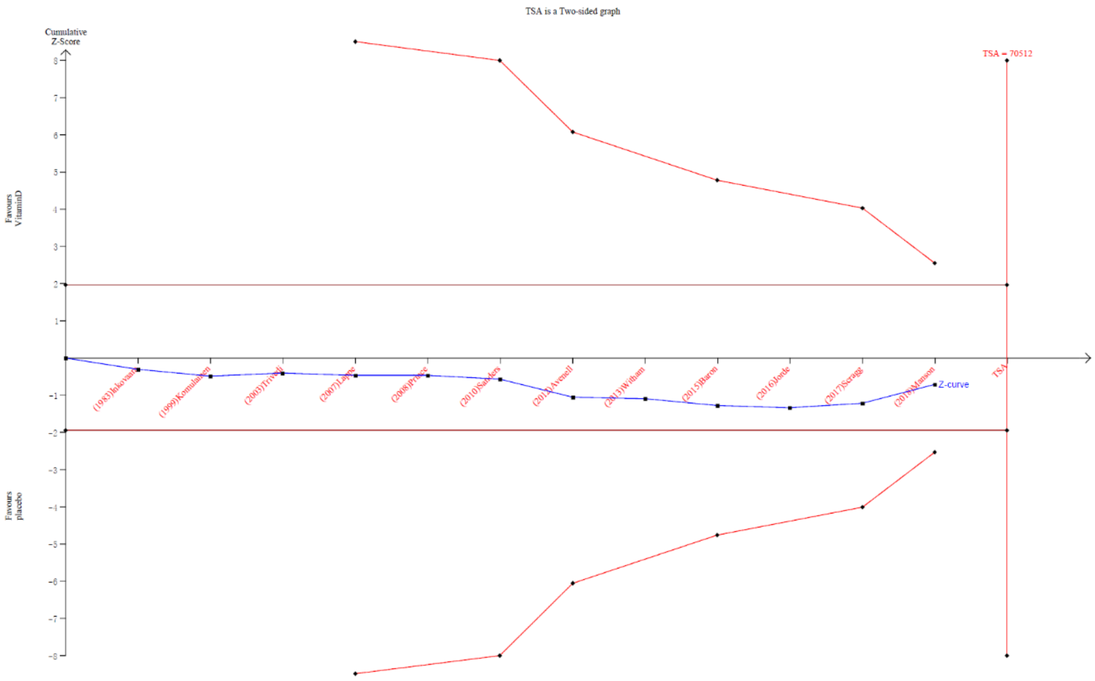

A diversity-adjusted information size (DIS) size of 70512 patients was calculated based on an anticipated relative risk reduction (RRR) of 15% (event proportion of 1.8% in the control arm, α=0.05 (two-sided), β=0.20 (power 80%)). The blue cumulative z-curve was constructed using a random-effects model and crossed the boundary for futility.

Figure S11: Trial sequential analysis for myocardial infarction, RRR=15%

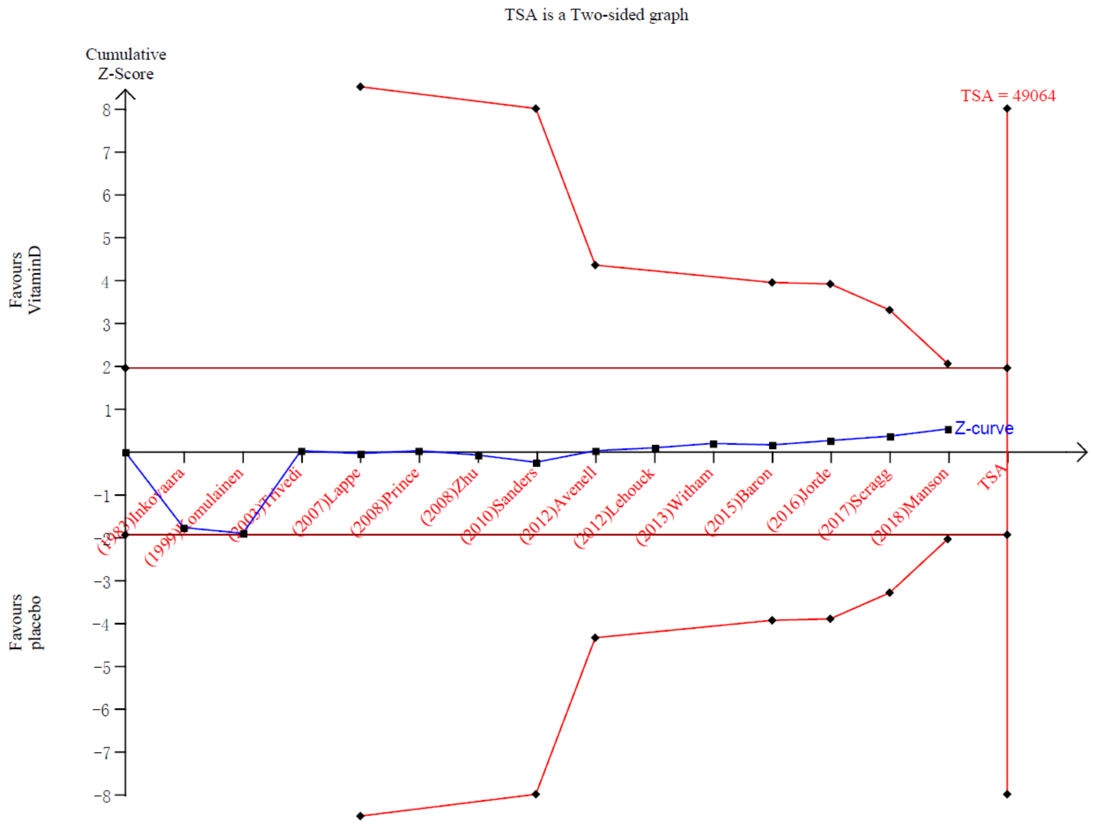

A diversity-adjusted information size (DIS) size of 49064 patients was calculated based on an anticipated relative risk reduction (RRR) of 15% (event proportion of 2.5% in the control arm, α=0.05 (two-sided), β=0.20 (power 80%)). The blue cumulative z-curve was constructed using a random-effects model and crossed the boundary for futility.

Figure S12: Trial sequential analysis for cardiovascular events,

RRR=15%

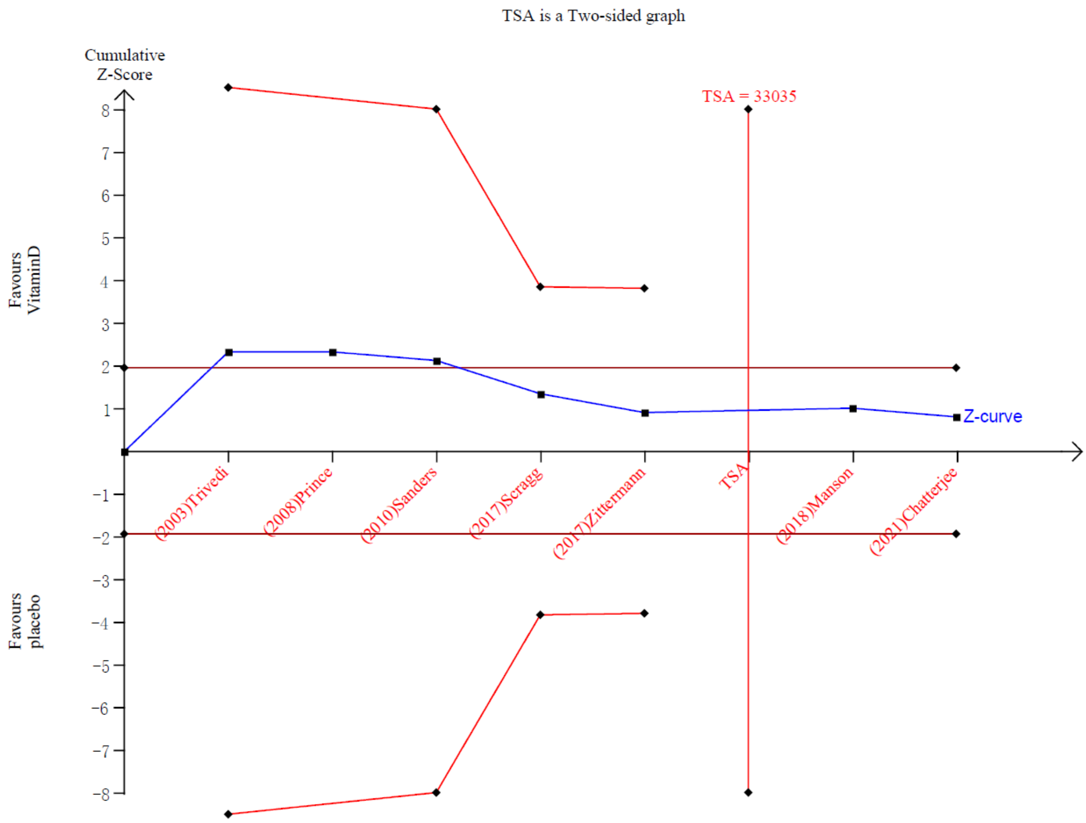

A diversity-adjusted information size (DIS) size of 33035 patients was calculated based on an anticipated relative risk reduction (RRR) of15% (event proportion of 6.8% in the control arm, α=0.05 (two-sided), β=0.20 (power 80%)). The blue cumulative z-curve was constructed using a random-effects model and crossed the boundary for futility.

Figure S13 Trial sequential analysis for cerebrovascular events, RRR=15%

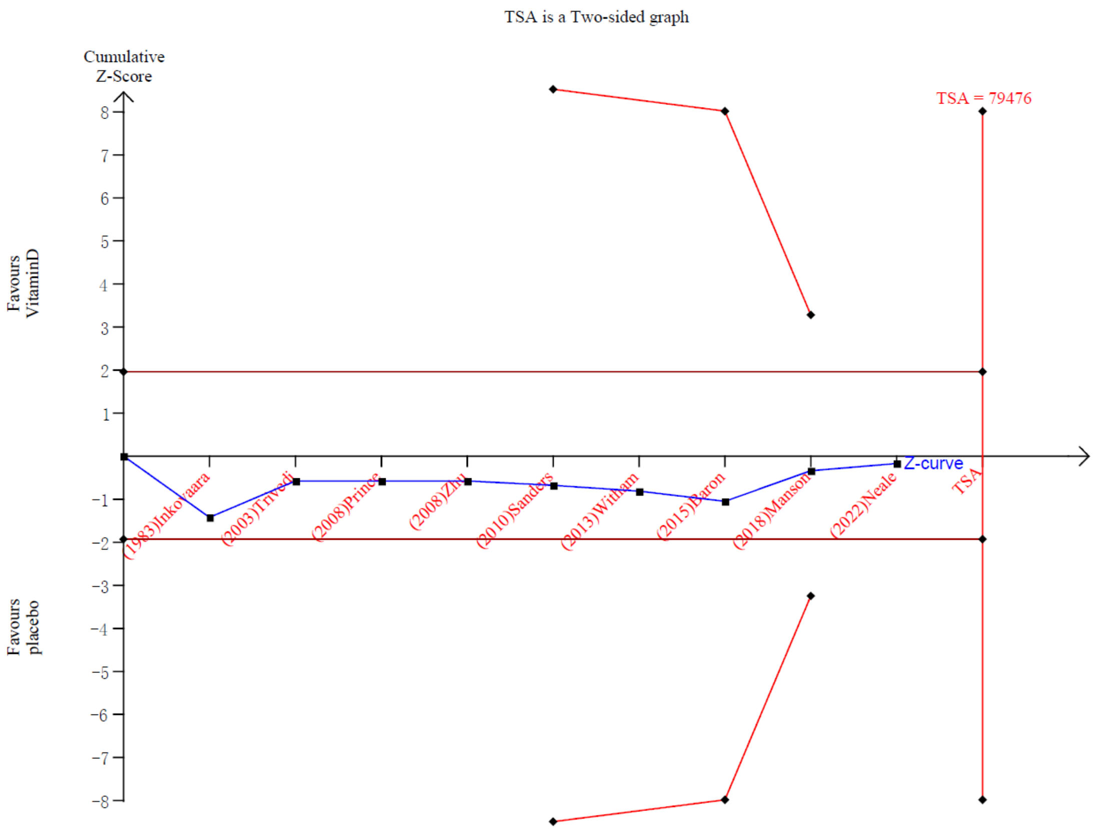

A diversity-adjusted information size (DIS) size of 79476 patients was calculated based on an anticipated relative risk reduction (RRR) of15% (event proportion of 1.6% in the control arm, α=0.05 (two-sided), β=0.20 (power 80%)). The blue cumulative z-curve was constructed using a random-effects model and crossed the boundary for futility.
